# Inhibition of a NEDD8 Cascade Restores Restriction of HIV by APOBEC3G

**DOI:** 10.1371/journal.ppat.1003085

**Published:** 2012-12-27

**Authors:** David J. Stanley, Koen Bartholomeeusen, David C. Crosby, Dong Young Kim, Eunju Kwon, Linda Yen, Nathalie Caretta Cartozo, Ming Li, Stefanie Jäger, Jeremy Mason-Herr, Fumiaki Hayashi, Shigeyuki Yokoyama, Nevan J. Krogan, Reuben S. Harris, Boris Matija Peterlin, John D. Gross

**Affiliations:** 1 Department of Pharmaceutical Chemistry, University of California, San Francisco, San Francisco, California, United States of America; 2 Graduate Program in Biophysics, University of California, San Francisco, San Francisco, California, United States of America; 3 Department of Medicine, University of California, San Francisco, San Francisco, California, United States of America; 4 Department of Biochemistry and Biophysics, University of California, San Francisco, San Francisco, California, United States of America; 5 Department of Biochemistry, Molecular Biology and Biophysics, Institute for Molecular Virology, Center for Genome Engineering, University of Minnesota, Minneapolis, Minnesota, United States of America; 6 Department of Molecular and Cellular Pharmacology, University of California, San Francisco, San Francisco, California, United States of America; 7 RIKEN Systems and Structural Biology Center, Tsurumi, Yokohama, Japan; 8 Department of Biophysics and Biochemistry, Graduate School of Science, The University of Tokyo, Hongo, Bunkyo-ku, Tokyo, Japan; 9 California Institute for Quantitative Biosciences, QB3, University of California, San Francisco, San Francisco, California, United States of America; 10 J. David Gladstone Institutes, San Francisco, California, United States of America; Duke University Medical Center, United States of America

## Abstract

Cellular restriction factors help to defend humans against human immunodeficiency virus (HIV). HIV accessory proteins hijack at least three different Cullin-RING ubiquitin ligases, which must be activated by the small ubiquitin-like protein NEDD8, in order to counteract host cellular restriction factors. We found that conjugation of NEDD8 to Cullin-5 by the NEDD8-conjugating enzyme UBE2F is required for HIV Vif-mediated degradation of the host restriction factor APOBEC3G (A3G). Pharmacological inhibition of the NEDD8 E1 by MLN4924 or knockdown of either UBE2F or its RING-protein binding partner RBX2 bypasses the effect of Vif, restoring the restriction of HIV by A3G. NMR mapping and mutational analyses define specificity determinants of the UBE2F NEDD8 cascade. These studies demonstrate that disrupting host NEDD8 cascades presents a novel antiretroviral therapeutic approach enhancing the ability of the immune system to combat HIV.

## Author Summary

The APOBEC3 family of editing enzymes catalyzes lethal hypermutation of retroviral genomes to block spread of virus in host. HIV Vif targets APOBEC3 family members for destruction by a cellular ubiquitin ligase containing CUL5. A major goal in the design of the next generation of antiretroviral therapies is to find an inhibitor of Vif so that the activity of the APOBEC3 family of antiretroviral enzymes can be restored. We define a three-enzyme cascade that is required to activate Vif by addition of the ubiquitin-like NEDD8 protein to CUL5. MLN4924, an anti-cancer compound currently in phase 1 clinical trials, inhibits the NEDD8 cascade, blocks the action of Vif, and thus has potent anti-HIV activity. Furthermore, our studies define downstream drug targets in the NEDD8 cascade more selective for inhibition of HIV Vif. We demonstrate pharmacological inhibition of HIV replication through a mechanism that restores the innate immunity provided by APOBEC3 enzymes by targeting a host pathway, providing additional candidates that could be further exploited for therapeutic development. Inhibition of this NEDD8 cascade alone, or in combination with existing antiretroviral drugs could prove to be a useful treatment for HIV.

## Introduction

HIV relies on extensive interactions with the host in order to co-opt cellular transcription, mRNA export, translation and ESCRT pathways [1], [2], [3], [4]. In addition, HIV must subvert the immune system to achieve a chronic infection [5]. The global landscape of human-HIV protein interactions was recently reported, identifying a network of host pathways that could be potentially exploited to block viral replication [6]. However, physical maps do not provide evidence for function, and the task of validating the interdependences of HIV on host pathways remains an outstanding challenge required before alternative therapeutic strategies can be considered.

The accessory proteins of HIV are considered to be prime targets because they often hijack the ubiquitin-proteasome pathway to downregulate restriction factors that would otherwise block the spread of virus in host [5]. For example, the APOBEC3 (A3) family of cytidine deaminases restricts retroviral replication to protect the infected host. When HIV lacks the viral infectivity factor (Vif), A3G and A3F enzymes are packaged into virions and perform lethal editing of viral cDNA, which occurs at the reverse transcription step [7], [8], [9], [10]. HIV Vif counteracts A3 enzymes by recruiting them to a Cullin-RING Ubiquitin Ligase (CRL) consisting of CUL5, a RING-box subunit (RBX), the canonical adaptor proteins Elongins B and C, and the recently described Vif-specific subunit core binding factor beta (CBFβ), which is normally involved in the control of transcription of RUNX genes [11], [12], [13], [14]. These subunits form the CRL5^Vif-CBFß^ holoenzyme, which acts in the last step of a three enzyme E1-E2-E3 cascade responsible for forming K48-linked polyubiquitin chains on APOBEC3 family members, targeting them for degradation by the 26S proteasome [11], [12], [15], [16], [17].

Covalent modification of a conserved lysine in the C-terminal domain of the Cullin subunit with the ubiquitin-like protein NEDD8 is essential for CRL function in metazoans [18]. This requires the action of a three-enzyme E1-E2-E3 cascade much like that of ubiquitin. NEDD8ylation activates a CRL, thus promoting the degradation of its substrates - a critical step in a broad array of cellular pathways including cell-cycle control, transcription, DNA repair and signaling [19]. As such, HIV Vif requires CUL5 NEDD8ylation to degrade APOBEC3G [11]. Given the broad dependencies of cellular protein homeostasis on CRL function, a potent mechanism-based inhibitor of the NEDD8 E1, MLN4924, was developed and found to be effective in suppressing tumor growth in xenograft models of cancer and is currently in phase 1 clinical trials [19], [20], [21].

In metazoans, there are parallel NEDD8 cascades wherein a single NEDD8 E1 charges the E2s, UBE2M and UBE2F, to promote NEDD8 conjugation of CRLs containing RBX1 or RBX2 respectively [22]. The RBX subunit is a critical determinant of cascade selection by making specific interactions with the NEDD8 E2 [22]. An important but unresolved question is the identity of the NEDD8 pathway responsible for activating the Vif-associated CRL responsible for A3G degradation. Early studies on the Vif-CUL5 complex implicated RBX1 as the RING subunit, since Vif co-immunoprecipitated with RBX1 in HIV-infected T-cells and overexpression of RBX1 or a mutant of CUL5 impaired in RBX1 binding had a dominant negative effect on Vif function [11]. However, subsequent studies of endogenous Cullin complexes suggested that CUL1-4 associate with RBX1, whereas CUL5 is normally in complex with RBX2 [22], [23]. In agreement, we recently found using tandem AP-MS that RBX2 is an integral part of the CUL5-Vif complex [6]. Here we define the NEDD8 cascade required for activation of HIV Vif and validate the concept that pharmacological inhibition of NEDD8 pathways can restore the restriction potential of the innate immune system. The pan-CRL inhibitor MLN4924 restores the restriction potential of A3G. The recently discovered NEDD8 conjugating enzyme UBE2F is the sole NEDD8 E2 necessary for Vif to counteract A3G, and the RING box protein RBX2 is required for Vif to promote spread of HIV in CD4+ T-cells. Structural and kinetic analysis of NEDD8 conjugation reveals how residues linking RBX2 to UBE2F impart specificity to the UBE2F NEDD8 cascade. These results advance our understanding of the activation of CRL5^Vif-CBFß^ by NEDD8 and suggest avenues by which Vif inhibition may be achieved.

## Results

### Pharmacological inhibition of the NEDD8 E1 with MLN4924 restores the restriction potential of A3G

**Figure 1:**
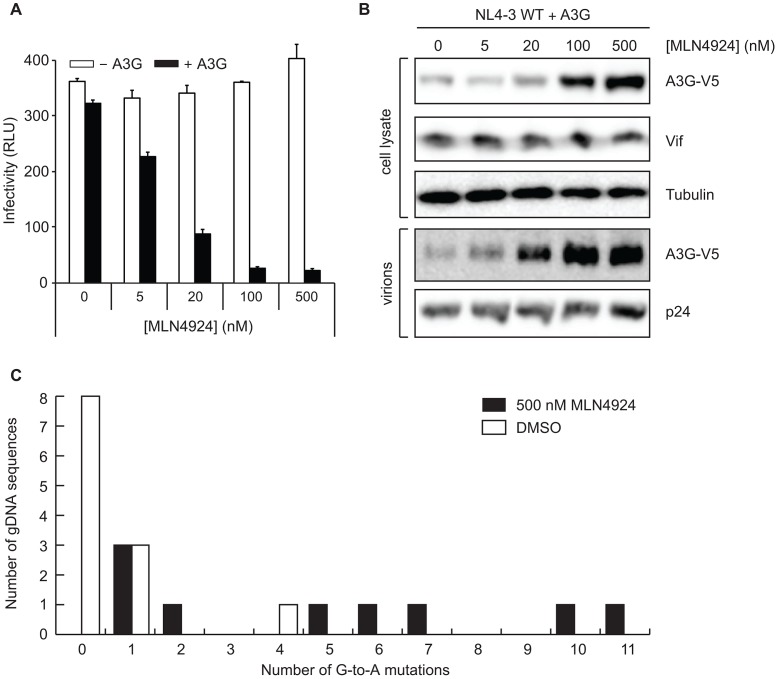
Pharmacological inhibition of NEDD8 E1 by MLN4924 blocks the ability of Vif to counteract A3G. **A,** Single-cycle infectivity assay of HIV_NL4-3_ produced in HEK293T cells transfected with empty vector control (white bars) or V5-tagged A3G (black bars, 120 ng) , 1 µg of NL4-3 proviral DNA and treated with indicated concentrations of MLN4924. **B,** Parallel immunoblots indicating MLN4924 restores steady-state levels of A3G in cells and packaging in virions. **C,** Quantitation of G to A mutations in gDNA sequences from virions produced in cells treated with either DMSO or 500 nM MLN4924.

Given the requirement of CUL5 NEDD8ylation for Vif activity, we reasoned that pharmacological inhibition of the NEDD8 modification would block Vif-mediated A3G degradation and cripple HIV infectivity. As proof-of-principle for the use of NEDD8 cascade inhibitors for antiretroviral therapy, we employed the recently described NEDD8 E1 inhibitor, MLN4924 [20]. A fully infectious molecular clone of HIV, HIV_NL4-3_, was co-transfected into HEK293 cells along with a mammalian expression construct containing A3G or empty vector. These cells were then treated with increasing concentrations of MLN4924, and the infectivity of resultant virus determined. Although increasing concentrations of MLN4924 did not significantly impact HIV infectivity in the absence of A3G (**Fig. 1A** **, white bars**), nanomolar concentrations of MLN4924 strongly reduced infectivity in the presence of A3G (**Fig. 1A** **, black bars**), indicating that MLN4924 impaired the ability of Vif to counteract APOBEC3G. Parallel immunoblots indicated that the compound impaired degradation of A3G and restored the ability of A3G to be packaged (**Fig. 1B**). Consequently and characteristic of A3G function, sequencing of viral genomic DNA from virus produced in the presence of MLN4924 revealed a significant increase in G to A mutations compared to virus produced in DMSO treated cells (**Figs. 1C** **, S1A**). It is important to note the dinucleotide context of the observed mutations, as A3G-dependent G-to-A mutations are thought to occur preferentially in a 5′GG context resulting in 5′AG, whereas mutations in the 5′ GA context occur by other mechanisms [9], [24], [25], [26], [27]. Consistent with this, the mutational context was 89% 5′GG (39) and 11% 5′GA (5) in the MLN4924-treated group, whereas it was 100% 5′GA (7) in the MLN4924-naïve group. Together, these data lend direct evidence as to how MLN4924 restored the restriction potential of A3G.

### Vif requires the NEDD8 E2 UBE2F to counteract A3G

**Figure 2:**
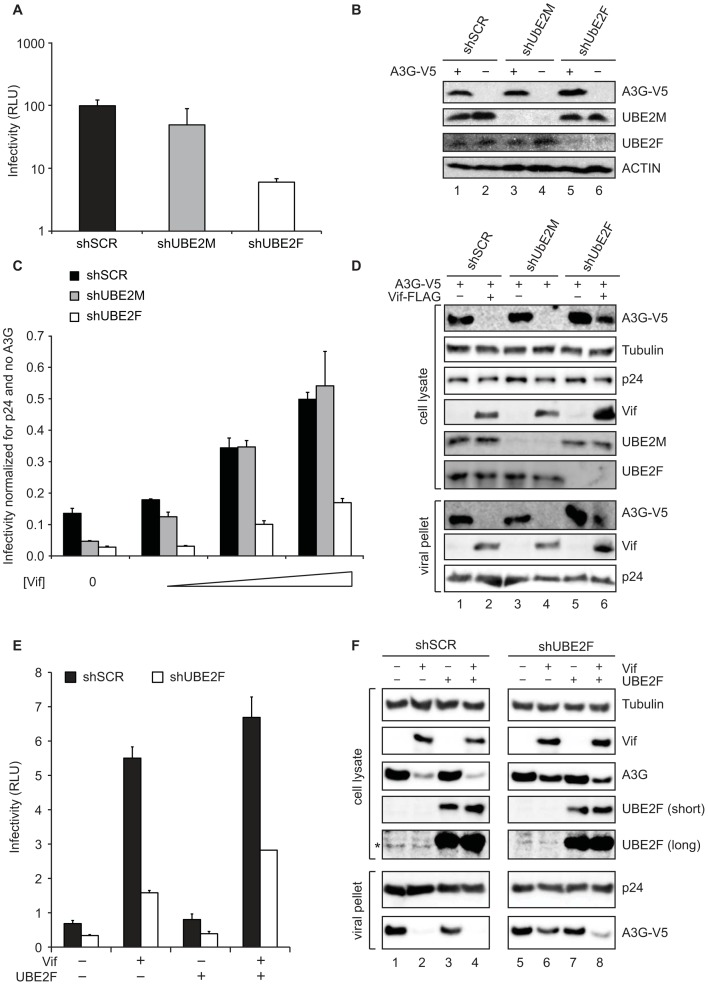
Vif requires the NEDD8 E2, UBE2F, to degrade A3G and mediate HIV infectivity. **A,** Knockdown of UBE2F but not UBE2M impairs viral infectivity. HEK293T cells stably depleted for UBE2M (grey bars), UBE2F (white bars) or in non-silencing control shRNA (black bars) were transfected with 1 µg proviral DNA (NL4-3), 340 ng A3G or empty vector and infectivity of produced virions was measured. Mean and 1 SD of duplicate experiments are graphed. **B,** Immunoblots corresponding to experiments shown in panel **A** show an increase in A3G stability in cells treated with UBE2F shRNAs relative to cells treated with UBE2M or scramble shRNAs. **C,** HIV requires Vif and UBE2F to fully neutralize A3G. Single-cycle infectivity assay of HIV_NL4-3_ΔVif produced in HEK293T cells stably depleted for UBE2M (grey bars), UBE2F (white bars) or non-silencing control shRNA (black bars). A3G (500 ng) or empty vector was co-transfected with 1 µg HIV_NL4-3_ΔVif and increasing amounts of Vif-FLAG (0, 15, 100, or 350 ng). Values were normalized to the infectivity in absence of A3G and mean and 1 SD of duplicate experiments are graphed. **D,** Immunoblots corresponding to samples in panel **C** without Vif and with the highest amount of Vif, indicating reduction of cellular and virally packaged A3G depends on Vif and UBE2F but not UBE2M. **E,** Infectivity of HIV_NL4-3_ΔVif produced in HEK293T cells stably depleted for UBE2F (white bars), or non-silencing control shRNA (black bars) in the presence of transfected HIV_NL4-3_ΔVif (1 µg), A3G-V5 (500 ng), Vif-FLAG (+Vif, 100 ng), RNAi-immune UBE2F–myc (+UBE2F, 10 ng)) or empty vector controls indicated by −Vif and −UBE2F. Mean and 1 SD of duplicate experiments are graphed. The presence of the RNAi-immune UBE2F vector in +Vif cells yielded a partial recovery of infectivity relative to control cells, but not in −Vif cells. Partial complementation may be due to the confounding effects of a mixed pool of knockdown cells and the observation that transfection of larger amounts of UBE2F inhibit virus production (data not shown). **F,** Immunoblots of cell lysates and virus particles corresponding to panel **E**. The ability of Vif to promote degradation and reduce packaging of A3G is strongly reduced in UBE2F KD cells (lanes 1, 2, 5, 6), and partially recovered in the presence of transfected RNAi-immune UBE2F (lanes 3, 4, 7, 8). The asterisk indicates the observed band for endogenous UBE2F. Long and short exposure times for the immunoblots are indicated.

Although inhibition of the NEDD8 E1 by MLN4924 potently restores the restriction potential of A3G, this compound inhibits NEDD8 conjugation of all Cullins and is toxic to CD4+ T-lymhocytes with a CT50 of 100 nM (**Fig. S1B**). We next sought to identify the NEDD8 cascade responsible for activating CRL5^Vif-CBFß^, reasoning that definition of downstream targets might allow for more selective inhibition. Initially, RBX1 was found to co-IP with Vif [11], but in a recent AP-MS study to identify new Vif-interacting partners we observed RBX2 in complex with Vif [12]. Since the identity of the RBX subunit determines NEDD8 E2 selectivity, we asked which NEDD8 E2 is responsible for Vif function and thus HIV infectivity in the presence of A3G [22]. Accordingly, we knocked down different NEDD8 E2s followed by co-transfection of HIV_NL4-3_ and A3G-V5 into HEK293T cells and performed single-cycle infectivity assays. Knockdown of UBE2F but not UBE2M or non-silencing control reduced infectivity by 10-fold with a concomitant increase in cellular A3G levels (**Fig. 2A,B**).

To systematically address the requirement of UBE2F for viral infectivity, experiments were performed with a Vif-deficient provirus (HIV_NL4-3_ΔVif) with transfected A3G and increasing concentrations of Vif-FLAG, following transduction with shUBE2F, shUBE2M or the non-silencing control shRNA (**Fig. 2C**). In the absence of Vif, virus infectivity was significantly impaired in all three knockdowns. With increasing amounts of Vif, knockdown of UBE2F blocked the ability of Vif to counteract A3G whereas non-silencing control and UBE2M knockdown had no effect on Vif function. Parallel immunoblot analyses revealed that knockdown of UBE2F impaired the ability of Vif to block viral packaging of A3G, consistent with the infectivity data (**Fig. 2D**). Additionally, the fraction of CUL5 containing the NEDD8 modification was reduced by knockdown of UBE2F, whereas knockdown of UBE2M had no effect on CUL5 NEDD8ylation in HEK293T cells (**Fig. S2**). The role of UBE2F was confirmed using an RNAi-knockdown and complementation strategy where expression of RNAi-immune UBE2F was able to partially restore the defect in viral infectivity observed when UBE2F was knocked down, decreasing the amount of APOBEC3G in cell lysates and packaged into virions (**Fig. 2E,F**). We conclude that NEDD8ylation of CUL5 by UBE2F is essential for viral infectivity.

### UBE2F stimulates polyubiquitin chain formation on APOBEC3G

**Figure 3:**
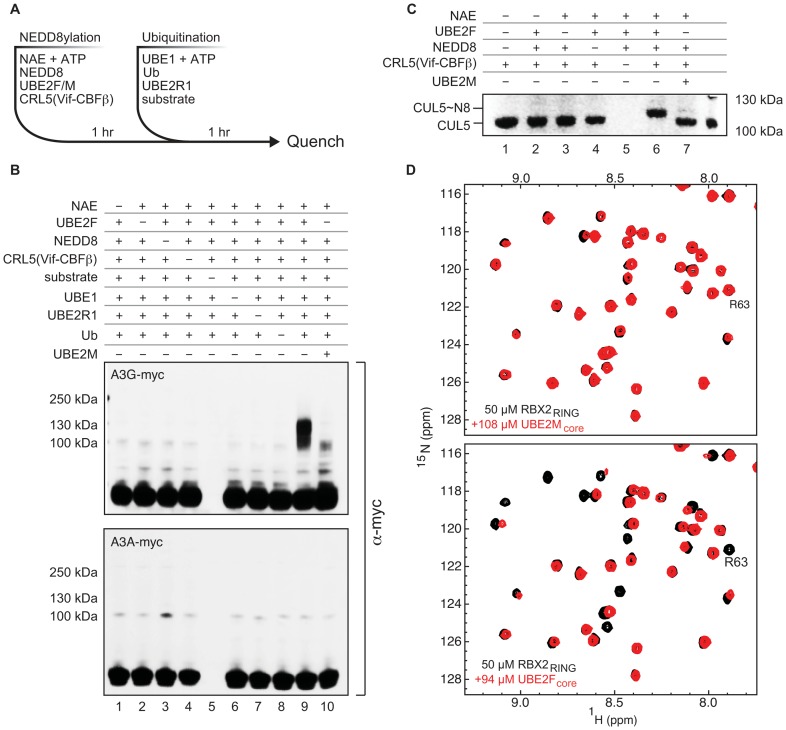
UBE2F is required for activation of CRL5^Vif-CBFß^ *in vitro*. **A,** Diagram of the ubiquitination protocol used in panel **B**. **B,** *In vitro* ubiquitination of A3G by recombinant CRL5^Vif-CBFß^ with UBE2R1 as ubiquitin conjugating enzyme requires UBE2F. Immunoblots of ubiquitination reactions containing myc-tagged A3G as the substrate show high-molecular weight polyubiquitin chains, require all protein components of the ubiquitin and NEDD8 activating systems and are only observed when UBE2F (lane 9) but not when UBE2M (lane 10) is used as NEDD8 conjugating enzyme. A3A is not susceptible to Vif and was used as a negative control. **C,** Coomassie-stained SDS-PAGE of NEDD8ylation “pulse” reaction indicates that under conditions used in panel **B** indicate CUL5 is completely NEDD8ylated by UBE2F; only a minor fraction (<5%) is NEDD8ylated by UBE2M. **D,** ^15^N-HSQC spectral overlays of RBX2_RING_ in the presence and absence of ∼100 µM, unlabeled full-length UBE2M (top) or UBE2F (bottom).

Previous studies indicate that formation of K48-linked ubiquitin chains on APOBEC3G is required for degradation and exclusion from virions and that recombinant purified CRL5^Vif-CBFß^ was able to catalyze synthesis of K48 linked chains on A3G [12], [17], [28]. Accordingly, we evaluated the effect of NEDD8 conjugation on activity of recombinant purified CRL5^Vif-CBFß^ as illustrated in **Fig. 3A**. We found that NEDD8ylation of CUL5 by UBE2F has a switch-like effect on formation of polyubiquitin chains on A3G but not the Vif-resistant deaminase A3A (**Fig. 3B**). An additional specificity control indicates this NEDD8-conjugated E3 ligase was also inactive in polyubiquitin chain formation on the Vif-resistant mutant of A3G (D128K, D130K) (**Fig. S3A,B**) [29]. In contrast, UBE2M only weakly affected activity of CRL5^Vif-CBFß^, which correlated with a low level of CUL5 NEDD8ylation (**Fig. 3B,C**). These observations are explained by ^15^N-HSQC NMR spectra showing UBE2F binds RBX2 but UBE2M does not (**Fig. 3D**). Consistent with the effect on viral infectivity, inhibition of the NEDD8 E1 by MLN4924 blocks charging of UBE2F with NEDD8, explaining why MLN4924 is able to restore the restriction potential of A3G (**Fig. S1C**). These results indicate UBE2F promotes activation of the polyubiquitin synthesis activity of CRL5^Vif-CBFß^ *in vitro*.

### RBX2 is required for replication of HIV in non-permissive CD4+ T-cells

**Figure 4:**
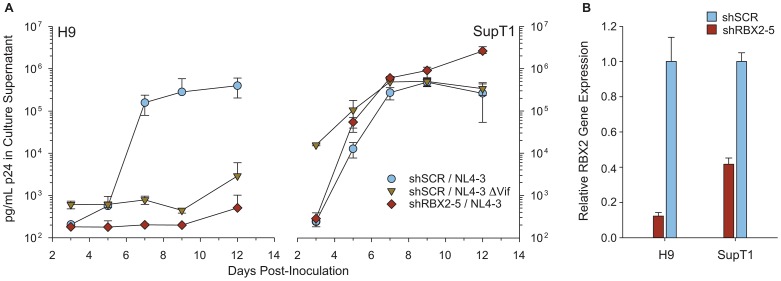
RBX2 is required for viral spread through non-permissive CD4+ H9 T-cells. **A,** HIV multiple round replication assays are shown for indicated viruses in Vif-permissive (SupT1) or Vif non-permissive (H9) cell lines transduced with viruses producing either control (shSCR) or RBX2-specific (shRBX2-5) shRNAs. Blue circles indicate shSCR treated cells infected with NL4-3 virus, yellow triangles indicate shSCR treated cells infected with NL4-3 virus lacking Vif, and red diamonds indicate shRBX2-5 treated cells infected with NL4-3 virus. Points are the mean of three independent biological replicates, error bars indicate 1 SD. **B,** Relative RBX2 mRNA knockdown normalized to non-silencing shSCR control as determined by RT-qPCR for the cell lines shown in panel **A.** Bars indicate the average of triplicate measurements, error bars are 1 SD.

We next evaluated the role of RBX2 in viral infectivity, since UBE2F is required for Vif function and previous studies indicate UBE2F forms a functional E2/RING pair with RBX2 [22]. Accordingly, we used shRNA to knockdown RBX1 or RBX2 and assayed for replication of HIV in non-permissive H9 or permissive SupT1 CD4+ T-cell lines that express high or very low levels of APOBEC3 restriction factors, respectively [30]. Although stable knockdown of RBX2 had no effect on HIV spread through permissive SupT1 cells, knockdown of RBX2 resulted in a sustained suppression of HIV spread through non-permissive H9 cells (**Fig. 4A,B**). Indeed, the degree of RBX2 knockdown correlated with increased suppression of HIV spread through non-permissive H9 cells (**Fig. S4)**. Transduction with non-silencing control shRNA did not hinder the replication of virus in either cell line (**Fig. 4A,B**). Culture supernatant HIV p24 antigen concentrations were commensurate with the fraction of cells positive for HIV antigen expression as determined by immunofluorescence assay (data not shown). Knockdown of RBX1 resulted in cell death in both H9 and SupT1 cells and precluded comparisons of viral replication. The observed toxicity of RBX1 knockdown is consistent with the established role of RBX1 as RING subunit of CRL1-4 [22]. In contrast, when RBX2 mRNA was reduced by more than 80%, greater than 90% of H9 cells were viable at 6 weeks post-transduction, as evidenced by trypan blue exclusion, and cells retained resistance to the stably integrated puromycin selective marker (data not shown). These findings are consistent with recent interaction and functional studies indicating RBX2 is the RING subunit of CRL5 [6], [22], [23]. We conclude that the NEDD8 cascade containing UBE2F and RBX2 is critical for activation of CRL5^Vif-CBFß^ and the ability of HIV Vif to counteract A3 restriction factors.

### Specificity determinants of the UBE2F/RBX2 NEDD8 cascade

**Figure 5:**
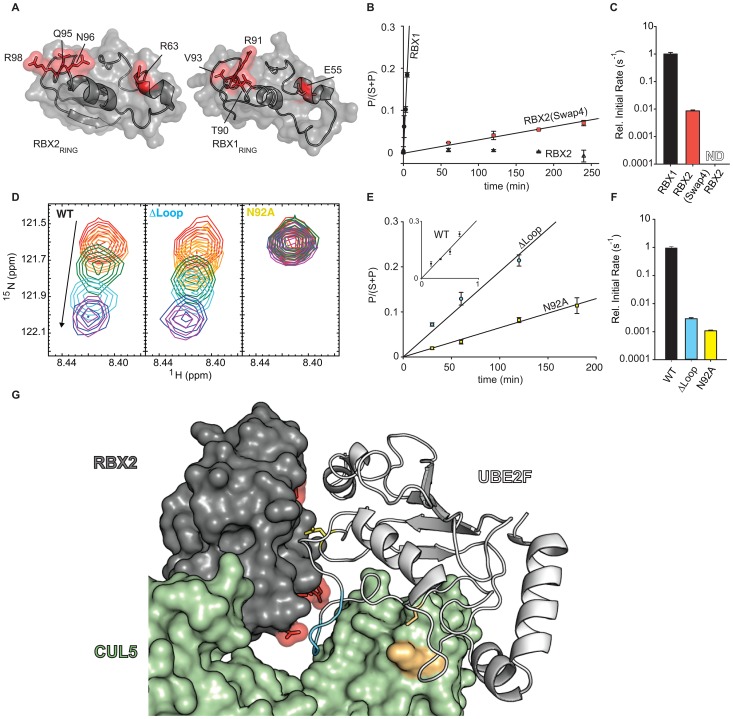
Structural basis for discrimination between NEDD8 pathways. **A,** Surface representations of RBX1_RING_ and RBX2_RING_ with divergent surface residues (Swap4) targeted for substitution analysis. Coordinates for RBX1, RBX2 were from PDB files 2EDI and 3DQV, respectively. **B,** Time courses of NEDD8 transfer from UBE2M to indicated CUL5/RBX complexes and **C,** relative initial rates for NEDD8ylation normalized to CUL5/RBX1. Error bars indicate standard deviation between at least two experiments. **D,** Excerpts from ^15^N-HSQC spectra of RBX2 titrated with increasing concentrations of unlabelled wild-type or mutant UBE2F. **E,** Time courses for NEDD8 transfer from wild-type and UBE2F mutants to CUL5/RBX2. Error bars indicate standard deviation between at least two experiments. **F,** Relative initial rates for NEDD8ylation of CUL5/RBX2 normalized by wild-type UBE2F. Error bars indicate standard deviation between at least two experiments. **G,** Model of CUL5/RBX2/UBE2F complex based on CUL1/RBX1 crystal structure (3RTR) and cIAP2/UBE2D2 crystal structure (3EB6) [33], [60]. UBE2F, RBX2 and CUL5 are shown in white, grey, and green respectively. The catalytic cysteine of UBE2F and the NEDD8 acceptor lysine of CUL5 (K724) are shown in orange; the unique loop insertion of UBE2F, blue; Asn92 of UBE2F, yellow sticks; and residues of RBX2 targeted for Swap4 mutation, red. Details of modeling can be found in the Experimental Procedures.

Previous domain-swapping experiments indicated the RING domain of the RBX subunit was a major specificity determinant for CRL conjugation by NEDD8 conjugating enzymes [22]. The structural basis for specificity is not well understood, especially since RBX1 and RBX2 are 50% identical in amino acid sequence. To understand in more detail how the RBX2 subunit of CUL5 discriminates between NEDD8 cascades, we used NMR chemical shift changes to map the binding interface and reveal divergent surface residues that could provide a basis for specificity. The binding surface mapped by NMR agrees well with other RING-E2 pairs, and guided our substitution analyses (**Fig. S3C, top**). We found two divergent surface regions centered on R63 and Q95 of RBX2 that line the interface with UBE2F, which is otherwise conserved with RBX1 (**Figs. S3C,D**). If these surfaces are important for conferring NEDD8 E2 specificity, then swapping residues from these regions between RBX1 and RBX2 should restore function of UBE2M with RBX2. An RBX2 mutant, RBX2(Swap4), was designed that swapped in four RBX1 residues to their equivalent positions in RBX2 (**Figs. 5A** **and S3E**). We compared conjugation of NEDD8 to CUL5/RBX2 or CUL5/RBX2(Swap4) and CUL5/RBX1 to evaluate the role of divergent RBX residues in conferring NEDD8 E2 specificity. Although CUL5/RBX1 complexes are not detected under physiological conditions, this heterodimer can be prepared by overexpression as described previously and serves as a positive control for NEDD8 conjugation by UBE2M [22], [23], [31], [32].

Conjugation of NEDD8 to CUL5/RBX1, CUL5/RBX2 or CUL5/RBX2(Swap4) was followed over time and revealed that UBE2M is able to conjugate CUL5/RBX1 but not CUL5/RBX2. Remarkably, CUL5/RBX2(Swap4) partially restored the ability of UBE2M to NEDD8ylate CUL5 (**Figs. 5B,C** **and S3F**). The ability of UBE2F to conjugate CUL5/RBX1 or CUL5/RBX2 was identical, and the Swap4 variant of RBX2 had only a small effect on the ability of UBE2F to conjugate CUL5 (**Fig. S3G–I**). These data indicate divergent residues of RBX2 block interactions with UBE2M and allow function with UBE2F.

Similar analyses of divergent surface features of UBE2F identified an active site proximal loop extension spanned by residues Ser124 to Gly129 (**Fig. S5A–C**). Deletion of this loop insertion in UBE2F (ΔLoop) reduced the rates of CUL5/RBX2 NEDD8ylation by more than 100-fold but did not affect binding to the RBX2 core domain as detected by NMR. In contrast, mutation of Asn92 of UBE2F to alanine blocked binding as observed by NMR and reduced activity by nearly 3 orders of magnitude, consistent with its predicted position on the UBE2F/RBX2 interface (**Figs. 5D–F** **, S5D,E**). HSQC NMR spectra indicate the mutants are folded (data not shown). A homology model of the CUL5/RBX2/UBE2F complex based on a recent crystal structure of the C-terminal domain of CUL1 in complex with RBX1 suggests the divergent loop of UBE2F may function to stabilize a catalytically active form of CUL5/RBX2/UBE2F that is poised for NEDD8 transfer (**Fig. 5G**) [**33**].

**Figure 6:**
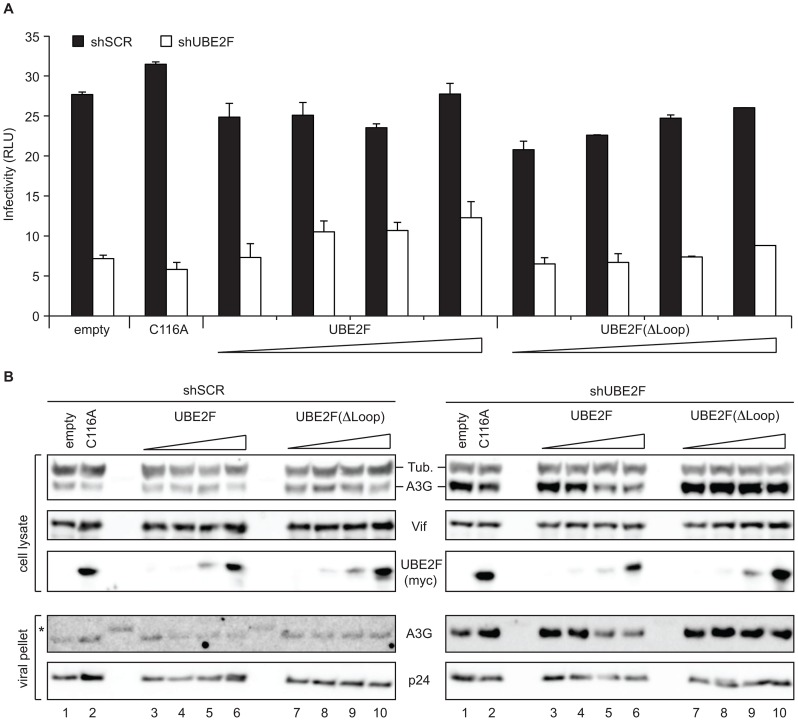
The UBE2F loop insertion is required for efficient viral infectivity. **A,** Single-cycle infectivity assay of HIV_NL4-3_ΔVif produced from HEK293T cells stably depleted for UBE2F (white bars), or non-silencing control shRNA (black bars) in the presence of transfected HIV_NL4-3_ΔVif (1 µg), A3G-V5 (500 ng), Vif-FLAG (100 ng), and increasing amounts of RNAi-immune wild-type or ΔLoop UBE2F-myc (0.2, 1, 3 or 10 ng), a catalytic mutant of UBE2F harboring a cysteine to alanine change at position 116 (10 ng), or empty vector control (10 ng). Mean and +-SD of duplicate experiments are graphed. **B,** Immunoblots of cell lysates and virus particles corresponding to panel **A**. A3G levels in cellular lysates and virus particles in UBE2F KD cells transfected with increasing amounts of RNAi-immune UBE2F (compare lanes 3–6, left and right), catalytic mutant UBE2F (C116A) or increasing amounts of UBE2F (ΔLoop) (lanes 1, 2, 7–10 left and right). To discern A3G levels in the virion in the shSCR lanes the immnoblots were exposed longer and non-specific bands from the protein ladder became apparent, as indicated by an asterisk.

To evaluate the requirement for the unique loop insertion of UBE2F for HIV infectivity, UBE2F was knocked down in HEK293T cells followed by transient transfection of HIV_NL4-3_ΔVif in the presence of A3G and Vif and increasing amounts of RNAi-immune plasmid encoding wild-type or UBE2F (ΔLoop). A catalytically dead UBE2F (C116A) that cannot be charged with NEDD8 was incorporated as a negative control. While UBE2F (C116A) was unable to rescue infectivity or A3G degradation by Vif, expression of wild-type UBE2F partially restored the defect in viral infectivity, when endogenous UBE2F was knocked down (**Fig. 6A,B**). Restoration of infectivity by wild-type UBE2F was dose-dependent and correlated with a decrease in cellular and virion packaged A3G as detected by immunoblot (**Fig. 6A,B**). In contrast and in line with our biochemical data, titration of UBE2F (ΔLoop) indicated that deletion of these UBE2F specific residues impaired its ability to complement UBE2F knockdown and restore infectivity which was reflected in the absence of A3G degradation **(** **Fig. 6A,B**). These results indicate the unique loop insertion of UBE2F is important for viral infectivity, consistent with the strong requirement of the loop for UBE2F activity *in vitro*. We conclude that the RBX2/UBE2F components of the metazoan specific NEDD8 cascade have evolved loop residues that act as specificity determinants during binding or promote the catalytic step, suggesting structural features that could be exploited for targeted pharmacological inhibition.

## Discussion

**Figure 7:**
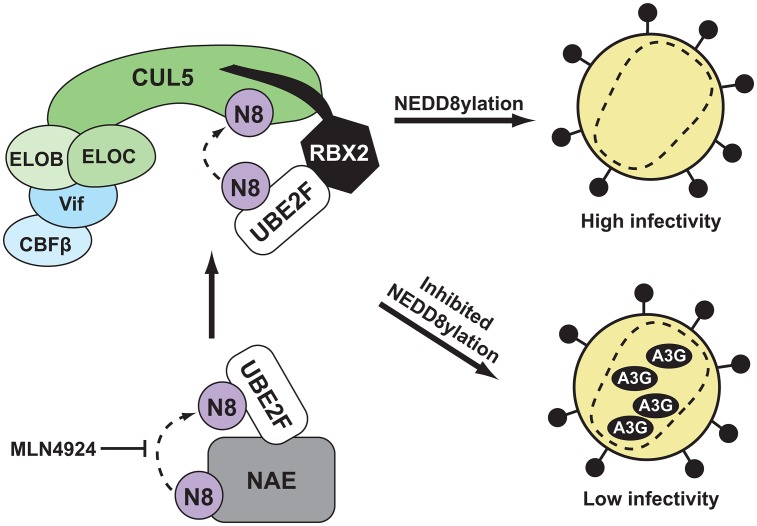
A model illustrating how inhibition of CUL5 NEDD8ylation leads to reduced infectivity of HIV. Two enzymatic steps must take place in order for CRL5^Vif-CBFß^ to be properly activated by NEDD8 conjugation, and therefore for A3G-degradation to take place, in cells infected with HIV. First, NEDD8 is loaded onto the E2 UBE2F by NAE. The small molecule MLN4924 is able to inhibit this step, blocking degradation of A3G and thereby reducing viral infectivity. Second, UBE2F is recognized by the RBX2 subunit of CRL5^Vif-CBFß^, and transfers NEDD8 to CUL5.

CRLs are estimated to affect turnover of 10% of cellular proteins, and hijack of this enzyme superfamily is a common viral strategy to evade the host immune response [20], [34]. Herein, we validate the concept that inhibition of NEDD8 cascades required to activate CRLs restores the innate immunity provided by restriction factors (**Fig. 7**). Pharmacological inhibition of the NEDD8 E1 by MLN4924 restored the ability of A3G to restrict HIV by disabling the Vif-hijacked E3 ligase, CRL5^VIF-CBFß^. Nanomolar concentrations of MLN4924 effected a strong increase in the amount of A3G detected in both HIV-infected cells and virions produced from these cells, resulting in significantly less infectious virions compared to those produced in untreated cells. Exposure of HIV to MLN4924 caused a significant increase in G to A mutations in the viral genomic DNA of progeny viruses, compared to inhibitor-naïve viruses, indicating that the loss in infectivity is due to A3G activity.

As MLN4924 inhibits NEDD8ylation of all CRLs, we asked which NEDD8 E2 and RING-subunit were responsible for activating CRL5^Vif-CBFß^ to potentially allow for more selective inhibition of NEDD8 conjugation. Such a strategy, if successful, would allow inhibition of Vif while minimizing the perturbation of protein homeostasis observed in cells treated with MLN4924 [20]. The RING subunit of CRLs designates the NEDD8 cascade and recent studies established CUL5/RBX2 is the functional heterodimer in cells [12], [22], [23]. Consistent with these observations, we find the NEDD8 conjugating enzyme UBE2F is required for HIV infectivity by acting as an essential activator of Vif-mediated degradation of A3G. Furthermore, we observed that RBX2 is critical for the replication of HIV in CD4+ T-cells expressing APOBEC3 restriction factors. These results are in line with previous work indicating that UBE2F and RBX2 are required for NEDD8ylation of CUL5 [22] and indicate the expanded NEDD8 cascade containing UBE2F is an important host pathway required for HIV infectivity.

Our *in vitro* studies suggest UBE2F is an essential cofactor for Vif likely because NEDD8 conjugation imparts a switch-like response on the polyubiquitin chain synthesis activity of the CRL5^Vif-CBFß^ ligase in a manner similar to that described for CRL1 (also known as the SCF) [35]. This effect requires specific interactions between UBE2F, RBX2, and CUL5 formed by residues at the periphery of the UBE2F-binding surface of RBX2, and a unique loop found in UBE2F that enhances NEDD8ylation activity for CUL5. The interactions identified by our combined NMR, mutational and kinetic analyses are consistent with a recent crystallographic study showing how NEDD8-E2 thioester may be positioned by the RBX subunit and the Cullin C-terminal domain (CTD) for efficient conjugation [33]. The unique active site proximal loop of UBE2F is important for viral infectivity and was found to contribute 2 log-units to the rate of NEDD8 conjugation but was dispensable for binding of RBX2. These observations are consistent the notion that the active site proximal loop of UBE2F could stabilize the transition state for catalysis, possibly by directly binding CUL5, but more detailed structural and kinetic studies will be required to prove this point.

The results presented here are at variance with the initial identification of RBX1 as the RING subunit of the Vif-CUL5 complex [11]. RBX1 was found to co-IP with Vif in HIV-infected T-cells, but the presence of RBX2 was not evaluated. Two experiments supported the function of RBX1 with Vif. Overexpression of a mutant CUL5(ΔRBX1) that blocked association with RBX1 was found to have a dominant negative effect on Vif function; however, this mutation disrupts a region of the CUL5 CTD that interacts with a strand conserved between RBX1 and RBX2 (**Fig. S3**) [36]; therefore, the effect of this mutant may derive from a loss of RBX2 as well as RBX1 binding. Additionally, it was shown that overexpression of RBX1 could inhibit Vif function, though RBX1 can interact with multiple ubiquitin and NEDD8 conjugating enzymes, so overexpression could titrate away E2 coenzymes required for endogenous CRL activity. We suggest the initial detection of RBX1 in Vif immunoprecipitates may result from copurified CUL2, which together with CUL5, was recently detected by affinity-purification mass-spectrometry studies of Vif expressed in Jurkat T-cells [6]. The observation that knockdown UBE2F or RBX2 impairs the ability of Vif to counteract APOBEC3G is in line with a body of work showing CUL5/RBX2 form a functional heterodimer in metazoans [22], [23], [37].

Prior studies indicate that NEDD8ylation of target CRL could be regulated, leading to the possibility that cofactors other than UBE2F and NEDD8 modulate Vif activity. In support of this idea, it was found that UBE2F activity was restricted to CUL5/RBX2 *in vivo* though it can function with RBX1 and RBX2 when overexpressed in cells or at high levels *in vitro* [22]. Negative and positive regulation of CRL activity can be achieved at the level of RING/E2 association as reported in recent studies. For example, Glomulin is an RBX1-specific binding factor that blocks association with ubiquitin conjugating enzymes thereby inhibiting chain formation activity of CRLs [38]. The yeast Defective in Cullin Nedd8ylation (DCN1) gene is required for the UBE2M homologue (UBC12) to promote NEDD8ylation of CUL1 (CDC53) [39]. Structural and functional studies indicate DCN1 and RBX subunits of the CRL work together as dual E3s to promote NEDD8 conjugation [40], [41]. An important challenge for future work is to understand RBX2 and UBE2F specific cofactors that modulate CRL5 activity and affect virus/host conflict through the Vif-APOBEC3 axis.

The Vif-APOBEC3 axis has long been considered an attractive drug target [42]. In terms of selectivity, direct and rational targeting of the interaction between Vif and APOBEC3 restriction factors would be ideal; however, high resolution structures of these complexes are unavailable and the absence of robust reconstituted systems for *in vitro* high-throughput screening have hampered this approach. Accordingly, most efforts to inhibit Vif function to date have utilized cell-based high throughput screens [43], [44]. Potentially promising lead compounds have been identified although selectivity and mechanisms of action are poorly understood [44], [45], [46]. MLN4924 represents an alternative approach to inhibiting Vif: it has a well-characterized mechanism of action; it is highly selective for the NEDD8 E1 (over Sumo and Ubiquitin UBE1); it is validated in mouse models of cancer and currently in clinical trials [20], [21]. It is tempting to speculate that MLN4924 might be useful for treating individuals infected with HIV and resistant to HAART but evaluation of efficacy and how this would be tolerated in the case of chronic infection remains a challenge for preclinical studies.

Alternatively, inhibition of UBE2F would block activation of CRL5 but not CRL1-4 and could therefore be a more selective route to Vif inhibition than MLN4924. Since Vif counteracts several A3 enzymes present in T-lymphocytes [5], [30], [47], selective targeting of UBE2F with small molecule inhibitors to reduce the activity of CRL5 could potentially unleash the restriction potential of A3 enzymes without perturbing the function of CRL1-4. The recent discovery of a specific allosteric small molecule inhibitor of a ubiquitin E2 (hCDC34a) suggests selective inhibition of NEDD8 conjugating enzymes is indeed possible [48]. Inhibition of host pathways required for HIV infectivity such as NEDD8 cascades, in isolation or in combination with current antiretroviral therapies, could be an important strategy to avoid resistance mutations and could be a viable antiretroviral therapy.

## Materials and Methods

### Stable shRNA knockdown of host factors in HEK293T cells and single-cycle infectivity assay

pLKO.I lentiviral vector plasmids expressing shRNA targeting UBE2M/F and a puromycin selectable marker were purchased from Open Biosystems: UBE2M: TRCN0000007259 ; UBE2F: TRCN0000034110. A control pLKO.I lentivector plasmid expressing a scramble shRNA was obtained from Addgene [49]. Lentiviral vectors pseudotyped with vesicular stomatitis virus glycoprotein G (VSV-G) were produced and normalized for p24 capsid content as previously described [50]. At day 1, HEK293T cells were transduced with shRNA vectors in 12-well plates for 36 hours. At day 3, transduced cells were replated in 6-well plates and stably transduced cells selected in 4 µg/ml puromycin (Invitrogen) for 72 hours. At day 5, transduced and selected cells were transfected with relevant plasmids to produce HIV_NL4-3_ΔVif or NL4-3-Luc reporter virus, in the presence or absence of exogenous APOBEC3G, and HIV Vif, using Fugene 6 transfection reagent (Roche). After 48 hours, virus was harvested and filtered using 0.45 µM filters (Millipore) to remove cell debris. TZMbl reporter (in case of HIV_NL4-3_ΔVif or WT virus) or GHOST (in case of NL4-3-Luc reporter virus) cells were infected with virus normalized for p24 capsid content as determined by p24 ELISA (Pierce). After 48 hours cells were lysed and luciferase activity determined using a Luciferase Assay Kit (Promega). To determine viral infectivity in the presence of MLN4924 (ActiveBiochem) , HEK293T cells were transfected for 24 hours with plasmid pNL4-3 WT before compound was added. After 24 hours, virus was harvested and TZMbl cells infected with virus normalized for p24 capsid content as determined by p24 ELISA (Pierce) for 6 hours. After 24 hours cells were lysed, and luciferase activity determined using a Luciferase Assay Kit (Promega). For analysis of APOBEC3G incorporation in the viral particles, virus was concentrated by sucrose cushion ultracentrifugation as described previously [10]. Plasmids expressing APOBEC3G-V5 and Vif-FLAG were described previously [10]. HIV_NL4-3_ΔVif was produced by deletion of the Vif start codon and introduction of tandem stop codons introduced downstream in the Vif gene of the HIV_NL4-3_ WT plasmid. All transfections were performed with the same total amount of DNA and were complemented with empty plasmid vector pcDNA3.1 when either Vif, A3G or both were withheld from the experiment.

### Determination of G to A mutation rate in viral genomic DNA

Viral gDNA mutation rates were determined as described previously by Russell et al. [51]. In short, virus was produced in the presence of A3G in HEK293T cells in the presence of 500 nM MLN4924 or DMSO. The resulting virus was used to infect TZMbl cells and after 36 hours viral gDNA was prepared using QIAGEN Genomic DNA Purification kit (Qiagen). HIV specific sequences were Taq PCR amplified using primers F: GTCTGTTGTGTGACTCTGGTAAC and R: CCTGTCTGAAGGGATGGTTGTAG and TOPO-TA subcloned (Invitrogen). Sequences were determined and the amount of G to A mutations counted in each viral sequence.

### Immunoblots

Immunoprecipitated complexes were separated by 15% SDS-PAGE and transferred to HyBond-ECL nitrocellulose membranes. Following blockage in a 5% milk TBS solution, membranes were probed with appropriate primary antibodies to FLAG-peptide (Sigma), V5 (R961-25, Invitrogen), Actin (Ab8227, Abcam), Myc (Ab9106, Abcam), tubulin (Ab4074, Abcam), UBE2F (15707, Abcam), UBE2M (Rockland Pharmaceuticals), RBX1 (Invitrogen), HIV p24 (Ab9071, Abcam). Appropriate secondary antibodies were applied and immnoblots were visualized by ECL.

### Ubiquitination assays

Ubiquitination assays were performed at room temperature with the ubiquitin activating system containing: 2 mM ATP, human ubiquitin activating enzyme (UBE1) (200 nM), wild-type ubiquitin or variants (methylated or K48R) (75 µM), 4 µM E2 (UBE2R1) in addition to 0.625 µM Vif E3 and 200 nM APOBEC3 proteins in buffer containing 30 mM Tris-Cl (pH 7.3), 100 mM NaCl, 5 mM MgCl_2_, in a total reaction volume of 10 ml. CRL5^Vif-CBFß^ was pre-NEDD8ylated in conditions that included: 50 mM NaCl, 50 mM Tris-Cl pH 7.6, 2.5 mM MgCl_2_, 2 mg/ml BSA, 2 mM ATP, 100 nM NEDD8 activating enzyme (NAE), 2 µM UBE2F, 30 µM NEDD8 and 4 µM Vif E3 ligase. After 1 hr, the NEDD8 reaction mixture was diluted ∼6 fold upon the addition of the ubiquitin activating system and substrate. The ubiquitination reactions were quenched after 1 hr by the addition of 2× SDS loading dye. UBE1, NEDD8, and ubiquitin variants were purchased from Boston Biochem. Ubiquitinated A3 proteins were detected using a monoclonal anti-c-Myc antibody (Sigma).

### Protein expression and purification

All constructs and mutations were generated by standard PCR and restriction-based cloning methods unless otherwise noted. HIS_6_-tagged UBE2F full–length or residues 26–185 (UBE2F_core_), GST-tagged UBE2M, HIS_6_-UBE2R1, HIS_6_-GB1-tagged RBX2_RING_ (44–113) were expressed in E. coli. HIS_6_-GB1-tagged CUL5-RBX2 was co-expressed in *E. coli* from a pRSF-Duet plasmid. All tags were removed by TEV protease or thrombin (Sigma). All proteins were subjected to size exclusion chromatography for the final purification. The RBX2(Swap4) mutant was obtained by custom cDNA synthesis (Gene Art). All constructs were verified by sequencing of the entire open reading frame.

For expression, plasmids encoding UBE2F, RBX2_RING_ and CUL5/RBX2, were transformed into either *E. coli* BL21(DE3)-Star or BL21(DE3) (Invitrogen) cells and grown at 37°C to an optical density of 0.4–0.6, incubated at 17°C until an optical density of 1.2 was reached and induced with 0.5 mM IPTG overnight. 100 µM ZnCl_2_ was added to cultures harboring RING-containing constructs approximately one hour prior to induction. Unlabelled, ^15^N- or ^13^C-labelled proteins were expressed in cells grown in LB media, M9 media supplemented with 1 gram of ^15^N NH_4_Cl, or M9 media supplemented with 2 grams of ^13^C-glucose, respectively.

Unless otherwise noted, all proteins were purified at 4°C according to the following protocol. 10 µM ZnCl_2_ was included in the buffers for all proteins containing a RING-domain. Cells from 1L of culture were resuspended in 20 mL lysis buffer (20 mM HEPES pH = 7.6, 500 mM NaCl, 10 mM imidazole, 0.1% NP-40, 10% glycerol, 10 mM β-mercaptoethanol, 1 mg/mL lysozyme (Sigma), 0.2 tablet EDTA-free complete protease-inhibitor cocktail (Roche)), and incubated on ice for 20 minutes followed by sonication and high-speed centrifugation. The soluble fraction was incubated with Ni-NTA resin (Qiagen) for 1 h, and loaded onto a gravity column. The resin was then extensively washed with at least 30 column volumes (CV) Lysis Buffer and 30 CV Wash Buffer (20 mM HEPES pH = 7.6, 500 mM NaCl, 20 mM imidazole, 10% glycerol 10 mM β-mercaptoethanol). Specifically bound proteins were eluted with 20 mL Elution Buffer (20 mM HEPES pH = 7.6, 500 mM NaCl, 250 mM imidazole, 10% glycerol 10 mM β-mercaptoethanol) per 5 grams cell pellet. Tags were removed by incubation with TEV protease overnight during dialysis against Dialysis Buffer (20 mM HEPES pH = 7.6, 500 mM NaCl, 10% glycerol, 1 mM DTT). Cleaved tags, uncleaved proteins, and TEV protease were removed by two passages through a Ni-NTA gravity column. Size exclusion chromatography into buffer (20 mM HEPES pH = 7.6, 150 mM NaCl, 1 mM DTT) was used as a final purification step. Proteins >95% pure were then concentrated and used as-is, or aliquoted into single-use portions and flash-frozen in liquid nitrogen. The NEDD8 E1 (NAE), NEDD8 containing an N-terminal PKA-site and GST-UBE2M were prepared as previously described [52]. Purified 6-protein CRL5^Vif-CBFb^ complex was obtained as described previously [12]. Recombinant A3A- and A3G-myc-His_6_ were purified from HEK293T cells as described [53], [54].

### NMR spectroscopy

All spectra were recorded at 20°C on Bruker spectrometers equipped with cryoprobes and processed with NMRPipe [55]. Backbone resonance assignments of UBE2F_core_ and RBX2_RING_ were obtained using standard triple resonance methodology [56]. Titration data were collected on a Bruker 800 MHz spectrometer outfitted with a cryoprobe using 50 µM ^15^N-labelled protein and increasing amounts of unlabelled ligand in a standard buffer (100 mM NaCl, 1 mM DTT and 25 mM HEPES, pH = 7.5). Chemical shift changes in titration series were analyzed using CCPNMR [57] and fit to standard ligand-binding curves in SigmaPlot.

### Stable knockdown of RBX1 and 2 in CD4+ T-cell lines and assay of viral spread

Stable knockdown of RBX1 or RBX2 in H9 and SupT1 CD4+ T-lymphoblastoid cell lines was performed via VSV-G pseudotpyed lentiviral transduction employing the pGIPz shRNAmir lentiviral vector system from Open Biosystems. Cells were obtained from the National Institutes of Health AIDS Reagent Program and maintained in RPMI + 11.5% fetal calf serum (Hyclone) at 37°C, 5% CO_2_. The following shRNAmir constructs were utilized: RBX1, V3LHS_637679 and V3LHS_637677; RBX2, V2LHS_197071, V3LHS_408994, and V3LHS_408992; Non-silencing scrambled control, RHS 4346. VSV-G psedutoyped lentiviruses were produced in HEK293T cells as described above for pLKO.I lentiviral vectors. Infectious titers (TU/mL) of pGIPz-derived pseudotyped lentiviruses were determined via serial dilution of virus over HEK293T cells followed by flow cytometric analysis for cellular green fluorescent protein (GFP) expression (visual indicator incorporated into the integrated pGIPz transgene cassette) 3 days following transduction. H9 and SupT1 cells were transduced with 20 TU/cell via centrifugal inoculation at 1200× g at 37°C for 2 hours in media containing 8 µg/mL polybrene. Input inoculum was then removed, the cells washed once with phosphate-buffered saline (PBS), and the media replaced. Five to seven days following transduction, when T-cells exhibited strong transgene-driven GFP expression, puromycin (Invitrogen) selection was initiated at 2 µg/mL for 7 days and afterward reduced at 0.5 µg/mL to maintain transgene expression. Knockdown efficiency was determined 14 days following the initiation of puromycin selection via RT-qPCR.

HIV_NL4-3_ and HIV_NL4-3_ΔVif (start codon deleted and tandem stop codons introduced downstream) were generated via transient transfection of HEK293T cells using Polyjet lipofection reagent (SignaGen) per manufacturer's protocol. Three days following transfection, virus-laden culture supernatant was harvested, DNAse-treated with 20 µg/mL DNAse (Roche), 0.45 µm filtered, aliquoted, and stored at −80°C. Virus titers were determined via p24 ELISA as described above.

HIV spreading assays were performed via inoculation of 250K transduced H9 or SupT1 T-cells with 25 ng of HIV (corresponding to a multiplicity of infection of 0.1) in 250 µL culture media in triplicate sets of wells in 96-well microtiter plates. Twenty-four hours post inoculation, input virus was aspirated, the cells washed with PBS, and the media replaced. The infections were then monitored in 48–72 hour intervals via immunofluorescence assay for cellular HIV antigen synthesis and via p24 ELISA in the culture supernatant for progeny virus production.

### RT-qPCR

Transduced cells were lysed with QIAshredder columns (QIAGEN) and total RNA was isolated using RNeasy Mini Kit (QIAGEN). On-column DNase digestion was performed using RNase-Free DNase Set (QIAGEN). Total RNA was reverse transcribed with iScript Reverse Transcription Supermix (BIO-RAD), using a mix of oligodT and random primers in a 20 µL reaction according to the manufacturer's protocol. Salt-free primers for RNAi target gene (RBX2: forward primer ACG TGG AGT GCG ATA CGT G; reverse primer ACA TTC TCC CCA GAC CAC AA, RBX1: forward primer TGC AGG AAC CAC ATT ATG GA; reverse primer GCG AGA GAT GCA GTG GAA GT) and reference gene (RPLP0: forward primer GCT GCT GCC CGT GCT GGT G; reverse primer TGG TGC CCC TGG AGA TTT TAG TGG) were generated (Integrated DNA Technologies) and cDNA levels were compared by quantitative PCR using SsoAdvanced SYBR Green Supermix (BIO-RAD) and CFX Connect Real-Time PCR Detection System (BIO-RAD). All reactions were performed in triplicate and individual samples were normalized to the human gene RPLP0. Relative gene expression was calculated using the ΔΔCq method as described by Livak and Schmittgen [58].

### NEDD8ylation assays

200 µM NEDD8 containing N-terminal PKA site was radiolabeled with 2,500 units of PKA (NEB) and 7 ml of ^32^P(γ)-ATP (specific activity of 6000Ci/mmol) in a total reaction volume of 50 ml of PKA reaction buffer (NEB). Pulse chase assays were performed at room temperature as previously described [22], with the following changes: the final concentration of E2 and CUL5 were 100 nM and 500 nM respectively. Reactions were visualized by phosphorimaging (Typhoon) after fractionation on 8–12% SDS-PAGE gels (Biorad), that were then dried and exposed to a storage phosphor screen overnight. Data were quantified as a ratio of product (NEDD8ylated CUL5) to the total of substrate and product (NEDD8ylated CUL5 and UBE2∼N8) using ImageQuant. Initial rates were determined as described previously [41].

Assays for inhibition of UBE2F charging were performed by pre-mixing NEDD8 E1 and MLN4924 in for 10 minutes, and adding this to a second solution to give a final concentration of 12 µM UBE2F, 5 nM NEDD8 E1, 1.5 µM ^32^P-labelled NEDD8, 50 µM ATP and a variable concentration of inhibitor in 10 µL of 1× reaction buffer (same as above). Reactions were stopped after 3 minutes by the addition of 1 equivalent of non-reducing SDS-PAGE loading dye containing 10 mM EDTA. Gels were visualized as above. Bands were quantified by comparison to a simultaneously exposed dilution series of a known concentration of ^32^P-NEDD8. Data from multiple independent experiments was fit to a 4-parameter logistic equation, yielding an IC50 of 38±4.3 n.

### Modeling

The model of UBE2F/RBX2 was created using NMR chemical shift perturbations as HADDOCK restraints using PDB codes 2ECL and 2EDI as inputs [59]. The model of CUL5/RBX2/UBE2F was generated using the ALIGN command of PyMol and PDB codes 2ECL, 2EDI, 3RTR and 3EB6 as inputs [33], [60].

## Supporting Information

Figure S1 **Effects of MLN4924 on A3G activity, cell viability and charging of UBE2F.** **A,** Alternative representation of data from **Fig. 1C** as percentages of sequences containing the indicated range of G to A mutations. **B,** Toxicity of MLN4924 in SupT11 CD4+ T-cell lines, a subclone of SupT1 [61]. The CT50 of MLN4924 was determined via serial dilution of compound over 250K SupT1 cells in triplicate sets of cultures. Residual cellular viability was determined via colorimetric (3-(4,5-Dimethylthiazol-2-yl)-2,5-diphenyltetrazolium bromide (MTT) metabolic assay after three days of cell culture with compound. Percent viable cells is calculated via comparison to drug-naïve (100% viable) and media (0% viable) controls. Error bars are 1 SD. The vertical dashed line denotes the 50% cellular viability value at 120 nM MLN4924. **C,** Charging of UBE2F by the NEDD8 E1 is inhibited *in vitro* by MLN4924. Formation of NEDD8∼UBE2F conjugates was monitored by following ^32^P-NEDD8 after incubation with NEDD8 activating system and E2. Percent inhibition is graphed, where error bars indicate the standard deviation for two independent experiments.
(EPS)

Figure S2 **Knockdown of UBE2F reduces CUL5 NEDD8ylation.** Immunoblots of CUL5 in extracts of virus producing HEK293T cells treated with shRNA for UBE2M, UBE2F and non-silencing control indicate the fraction of NEDD8ylated CUL5 is reduced by shUBE2F but not shUBE2M or non-silencing control.
(EPS)

Figure S3 **Specificity of** CRL5^Vif-CBFß^ **ubiquitin ligase for substrate and NEDD8 conjugating enzymes.** **A,** *In vitro* ubiquitination of A3G by recombinant CRL5^Vif-CBFß^ is blocked in the double-mutant A3G(D128K,D130K). Immunoblots of ubiquitination reactions containing myc-tagged wild-type or mutant A3G as the substrate and using wild-type and ubiquitin variants (Me-Ub or K48R) are shown. The pattern for Me-Ub and K48R is similar, consistent with previous studies showing CRL5^Vif-CBFß^ can form K48 chains on Vif susceptible A3 substrates when UBE2R1 is used as E2 [12]. The asterisk indicates a non-specific band present in preparation of A3 proteins. **B,** Coomassie-stained SDS-PAGE of NEDD8ylation “pulse” reaction indicates that under conditions used in panel **A** indicate CUL5 is completely NEDD8ylated in the reactions shown. **C,** RBX2_RING_ is shown, colored by chemical shift perturbation upon addition of UBE2F_core_ (top), and including RBX1/2 conservation data, as cyan surface (bottom). **D,** Conservation between RBX2 (top) and RBX1 (bottom) is mapped in cyan onto the respective NMR or crystal structure. **E,** Plot of composite chemical shifts upon addition of UBE2F_core_ to RBX2_RING_, as calculated by √((δH_apo_−δH_bound_)2+((δN_apo_−δN_bound_)/5)^2^). Horizontal lines indicate the mean chemical shift perturbation (black) or the mean plus one standard deviation (red). Columns colored cyan indicate conservation between RBX1 and RBX2. Green coloration indicates positions chosen for swap mutations made in RBX2(Swap4). Assigned secondary structure and domain organization is shown above. Coordinates for UBE2F, RBX1 and RBX2 were derived from 2EDI, 3DQV and 2ECL respectively. **F,** Raw kinetic data from pulse-chase NEDD8ylation experiments following transfer of ^32^P-labelled NEDD8 from UBE2M onto various constructs as graphed in Fig. 5B. **G,** Kinetic data from pulse-chase NEDD8ylation experiments following transfer of ^32^P-labelled NEDD8 from UBE2F onto CUL5/RBX1, CUL5/RXB2 and CUL5/RBX(Swap4) indicating the Swap4 substitution does not significantly affect function with UBE2F. **H,** Plots of data from **G**. (CUL5/RBX1, black circles; CUL5/RBX2(Swap4), green triangles; CUL5/RBX2, white squares) Error bars indicate standard deviation of measured P/(S+P) ratios for at least two independent experiments. **I,** Relative initial rates for NEDD8ylation are shown as fit by solid lines in **H**. Values are normalized to the rate of CUL5-RBX1. Error bars indicate standard deviation between at least two experiments.
(EPS)

Figure S4 **RBX2 knockdown efficiency has a reciprocal correlation with the ability of HIV to spread through non-permissive H9 T-cells.** **A,** Spreading assays are shown for HIV_NL4-3_ viruses in Vif non-permissive (H9) cells stably expressing non-silencing shSCR control (blue circles) or RBX2-specific shRNAs (green triangles, red squares, and purple diamonds). Points are the average of three independent biological replicates, error bars are 1 SD. **B,** Relative RBX2 mRNA expression determined by RT-qPCR is shown for the cell lines shown in **A.** Error bars indicate the standard deviation calculated as described in Materials and Methods. **C,** shRNA targeting RBX2 does not have an off-target effect on RBX1. Relative RBX1 gene expression determined by RT-qPCR is shown for the cell lines shown in **Fig. 4A** **,** indicating expression level of RBX1 mRNA in H9 or SupT1 cells treated with RBX2-specific shRNAs. The replication delay observed in H9 cells knocked-down for RBX2 is not due to adventitious knockdown of RBX1, as little to no change in RBX1 mRNA levels was observed in H9 or SupT1 cell lines stably expressing shRBX2-5 relative to the control. Error bars indicate the standard deviation calculated as described in Materials and Methods.
(EPS)

Figure S5 **NMR analysis of UBE2F-RBX2 binding.** **A,** A model of the RBX2_RING_-UBE2F_core_ complex is shown, based on chemical shift-derived restraints. The model was created using the HADDOCK software package [59]. The RBX2_RING_ surface is shown in white and UBE2F_core_ is shown as a cartoon colored by chemical shift perturbation upon addition of RBX2_RING_. Low and high chemical shift perturbations correspond with grey and red coloration respectively. The Cys116 side chain of UBE2F is shown in stick representation, with a yellow sphere representing the sulphur atom. **B,** HSQC spectral overlays are shown for UBE2F_core_ alone (black), or with the addition of 384 µM RBX2_RING_ (red). **C,** Plot of composite chemical shifts upon addition of RBX2_RING_ to ^15^N-labelled UBE2F_core_, as calculated by √((δH_apo_−δH_bound_)2+((δN_apo_−δN_bound_)/5)^2^). Horizontal lines indicate the mean chemical shift perturbation (black) or the mean plus one standard deviation (red). Columns colored blue indicate the perturbed loop residues, **_124_**SIDGTG_129_. Blue coloration indicates the unique loop residues, and yellow indicates Asn96. Assigned secondary structure is shown above. **D,** Raw time course data for **Fig. 5E** **,** as described in main text. **E,** Quantification of UBE2F/RBX2 dissociation constant by NMR titrations. Data points and fitted curves are shown for the titration of 15N-labelled RBX2_RING_ with either UBE2F (black) UBE2F(ΔLoop) (blue) or UBE2F(N92A) (yellow). The D48 resonance is followed, as it is less broadened than more significantly perturbed residues in the interface.
(EPS)
